# Paget–Schrotter Syndrome in the Primary Care Setting: A Case Report and Scoping Review of Clinical Presentation and Diagnostic Delay

**DOI:** 10.3390/clinpract16090172

**Published:** 2026-09-19

**Authors:** Mohammed Hteit, Raein Ghazvinian, Mohammed Abdulrasak

**Affiliations:** 1 Blomman Vårdcentral (Primary Care Center), 212 14 Malmo, Sweden; mohammed.hteit@blommanvardcentral.se; 2 Department of Internal Medicine, Trelleborg Hospital, 231 25 Trelleborg, Sweden; raein.ghazvinian@skane.se; 3 Department of Clinical Sciences, Lund University, 221 00 Malmo, Sweden; 4 Department of Gastroenterology and Nutrition, Skane University Hospital, 214 28 Malmo, Sweden

**Keywords:** Paget–Schrotter syndrome, effort thrombosis, upper extremity deep vein thrombosis, primary care, diagnostic delay, thoracic outlet syndrome, PRISMA-ScR

## Abstract

**Background/Objectives:** Paget–Schrotter syndrome (PSS), or primary axillary-subclavian vein thrombosis, is an uncommon but clinically important form of upper extremity deep vein thrombosis. It often affects young, physically active patients and may initially be interpreted as a musculoskeletal disorder. This scoping review, combined with a contemporary primary care case, aimed to map the reported clinical presentation, diagnostic delay, and initial misdiagnosis of classic PSS. **Methods:** The review was conducted using the Arksey and O’Malley framework and reported in accordance with the PRISMA extension for scoping reviews (PRISMA-ScR). PubMed was searched for English-language publications from 1 January 2000 to 1 March 2026. Eligible reports described effort-induced thrombosis involving the axillary and/or subclavian venous segment. Secondary upper extremity deep vein thrombosis, non-effort thrombosis, pseudo-PSS, isolated distal venous thrombosis, and reports without extractable patient-level presentation data were excluded. Data were charted descriptively. **Results:** Among 1954 screened records, 143 studies met inclusion criteria and contributed 165 published patients. Together with the present case, the synthesis included 166 patients. Median age was 28 years, and 73.5% were male. Arm swelling (95.2%), pain (80.1%), cyanosis or discoloration (69.9%), and venous distension (70.5%) were the most frequent presenting features. Diagnostic delay was reported in 27.3% of patients with reported data, whereas initial misdiagnosis was reported in 21.7% of included patients. Musculoskeletal injury or muscle strain was the most common initial misdiagnosis, accounting for 66.7% of misdiagnosed cases. Pulmonary embolism was reported in 24.7% of patients, although this reported frequency is likely influenced by case-report publication bias, selective imaging, and preferential reporting of more severe presentations, and should not be interpreted as a population-level risk estimate. **Conclusions:** PSS should be considered in young or otherwise low-risk patients with unilateral arm swelling after strenuous or repetitive upper extremity activity. Greater awareness of this clinical presentation in primary care and general emergency settings may reduce diagnostic delay and post-thrombotic morbidity.

## 1. Introduction

Paget–Schrotter syndrome (PSS), also termed effort thrombosis or primary axillary-subclavian vein thrombosis, is a rare but clinically important subtype of upper extremity deep vein thrombosis (UEDVT) [1,2]. The condition is most often described in young and otherwise healthy individuals after repetitive, strenuous, or overhead upper extremity activity. It is generally considered the venous manifestation of thoracic outlet syndrome [1,2]. The pathophysiology of Paget–Schrotter syndrome can be understood within the framework of Virchow’s triad. Repeated compression and microtrauma of the axillary-subclavian venous segment between the clavicle and first rib may cause endothelial injury and venous stasis, thereby promoting thrombus formation [1,3].

Although PSS is uncommon, delayed recognition can have important consequences. Reported complications include pulmonary embolism, recurrent thrombosis, chronic venous obstruction, and post-thrombotic syndrome, with persistent pain, heaviness, swelling, venous collaterals, and reduced upper limb function [2,3,4]. Early identification is therefore clinically relevant, particularly because definitive imaging and treatment may be delayed when symptoms are attributed to more common benign conditions.

This diagnostic challenge is partly explained by the patient profile and symptom pattern. Many patients are young, active, and have no conventional venous thromboembolism risk factors. Symptoms such as arm pain, shoulder discomfort, heaviness, and exertion-related functional limitation may resemble muscle strain, tendon injury, or overuse syndromes. However, unilateral arm swelling, cyanotic discoloration, venous congestion, and visible collateral veins after upper extremity exertion should prompt consideration of UEDVT even in low-risk patients [1,3].

This issue is particularly relevant to primary care physicians and emergency clinicians, who are often the first point of contact. Most previous reviews of PSS have focused on treatment, including anticoagulation, catheter-directed thrombolysis, thoracic outlet decompression, and endovascular management [1,3,4]. Comparatively less attention has been given to the early clinical presentation, diagnostic pathway, and frequency of initial misdiagnosis. To address this knowledge gap, we combined a contemporary primary care case with a scoping review focused on clinical presentation, diagnostic delay, and early diagnostic recognition in classic PSS.

## 2. Materials and Methods

This study combined a case report with a scoping review. The review followed the methodological framework proposed by Arksey and O’Malley [5] and was reported in accordance with the PRISMA extension for Scoping Reviews (PRISMA-ScR) [6]. The corresponding checklist is available in Supplementary Table S1. The review aimed to characterize the reported clinical presentation, diagnostic pathways, diagnostic delay, and initial diagnostic impressions in patients with classic Paget–Schrotter syndrome.

A contemporary Swedish primary care case was included to illustrate the clinical features and diagnostic challenges identified in the reviewed literature. Written informed consent for publication of the case details and accompanying clinical photograph was obtained from the patient. Identifying information was minimized where possible. As the study consisted of a single anonymized case report combined with a review of the previously published literature, formal ethical approval was not required according to local regulations.

PubMed was searched on 16 March 2026 for English-language publications published between 1 January 2000 and 1 March 2026. PubMed was selected because the aim of this scoping review was to identify and characterize published case reports and case series describing the clinical presentation, diagnostic delay, and initial diagnostic assessment of classic Paget–Schrotter syndrome. The search strategy used free-text terms related to Paget–Schrotter syndrome and effort thrombosis and was designed to capture the multiple synonymous and historical terms used for the condition in the clinical case literature. No controlled vocabulary terms were used. The final PubMed search string was: (“Paget–Schrotter Syndrome” OR “Paget-von Schroetter Syndrome” OR “effort thrombosis” OR “primary axillary-subclavian vein thrombosis” OR “primary subclavian vein thrombosis” OR “effort-induced thrombosis”).

Reference lists of relevant publications were consulted when necessary to clarify eligibility or obtain additional patient-level information; however, a systematic hand-search of reference lists was not performed.

Studies were eligible if they reported patient-level data describing effort-induced thrombosis involving the axillary and/or subclavian venous segment consistent with classic Paget–Schrotter syndrome. Case reports and case series were included when information regarding presentation, diagnostic pathway, delay, or initial diagnosis could be extracted. Reports describing secondary upper extremity deep vein thrombosis, catheter-associated thrombosis, malignancy-associated thrombosis, non-effort thrombosis, pseudo-Paget–Schrotter syndrome, isolated distal upper extremity thrombosis, or thrombosis outside the axillary-subclavian venous segment were excluded. Review articles without extractable patient-level data, non-English publications, and reports for which full text could not be obtained were also excluded.

Titles, abstracts, and full texts were screened for eligibility according to the predefined criteria by the authors (M.H. and M.A.). Potentially eligible studies and cases with uncertainty regarding eligibility or classification of Paget–Schrotter syndrome were reviewed jointly, and disagreements were resolved through discussion and consensus. In studies reporting multiple patients, only cases meeting the definition of classic Paget–Schrotter syndrome were included. Reports describing mixed populations were excluded unless eligible cases could be separated from secondary or non-classic forms of upper extremity deep vein thrombosis. The study selection process was documented using a PRISMA flow diagram. A total of 1954 records were identified through PubMed searching. Following title and abstract screening, 180 articles underwent full-text assessment, of which 37 were excluded, resulting in 143 included publications.

Data were extracted by the first author (M.H.) using a standardized Microsoft Excel for Microsoft 365 (Version 2608; Microsoft Corporation, Redmond, WA, USA) form. Extracted data and cases with uncertainty regarding classification or variable assignment were reviewed in consultation with M.A. to ensure consistency and completeness of the final dataset. Information regarding patient demographics, provoking activities, anatomical predisposition, thrombophilia status, presenting symptoms and signs, diagnostic pathway, initial diagnosis, diagnostic delay, complications, and outcomes was collected whenever available. Treatment-related variables were also extracted as descriptive and contextual information to characterize the management strategies reported in published cases; however, these variables were not considered primary outcomes of the review. The contemporary case was incorporated into the final synthesis dataset as patient 166. For variables with incomplete reporting, percentages were calculated using variable-specific denominators, and absent findings were distinguished from non-reported data. Diagnostic delay was defined as a delay occurring after the patient’s initial healthcare contact and was distinguished from patient delay (i.e., the interval from symptom onset to first healthcare presentation). Initial misdiagnosis was defined as an explicitly reported alternative diagnosis or diagnostic pathway preceding the correct diagnosis of Paget–Schrotter syndrome.

A descriptive synthesis was performed. Categorical variables are presented as frequencies and percentages, whereas continuous variables are summarized using median and interquartile range when appropriate. Given the predominance of case reports and small case series, no meta-analysis was undertaken. The review was intended to map reported clinical patterns rather than provide population-level estimates of incidence, prognosis, or treatment effect.

Generative artificial intelligence (GenAI) tools were used during manuscript preparation to assist with the creation of the PRISMA flow diagram, graphical summary figure, and formatting of summary tables.

## 3. Case Presentation

A previously healthy 25-year-old woman presented with acute onset of swelling, pain, heaviness, and cyanotic discoloration of the right upper extremity after intensive CrossFit training involving repetitive upper-extremity exertion. She had no personal or family history of venous thromboembolism and no identifiable thrombotic risk factors, including smoking, oral contraceptive use, malignancy, recent immobilization, or known thrombophilia. She also reported a sensation of arm tightness and discomfort, with visible collateral venous distension over the shoulder and anterior chest wall.

**Figure 1:**
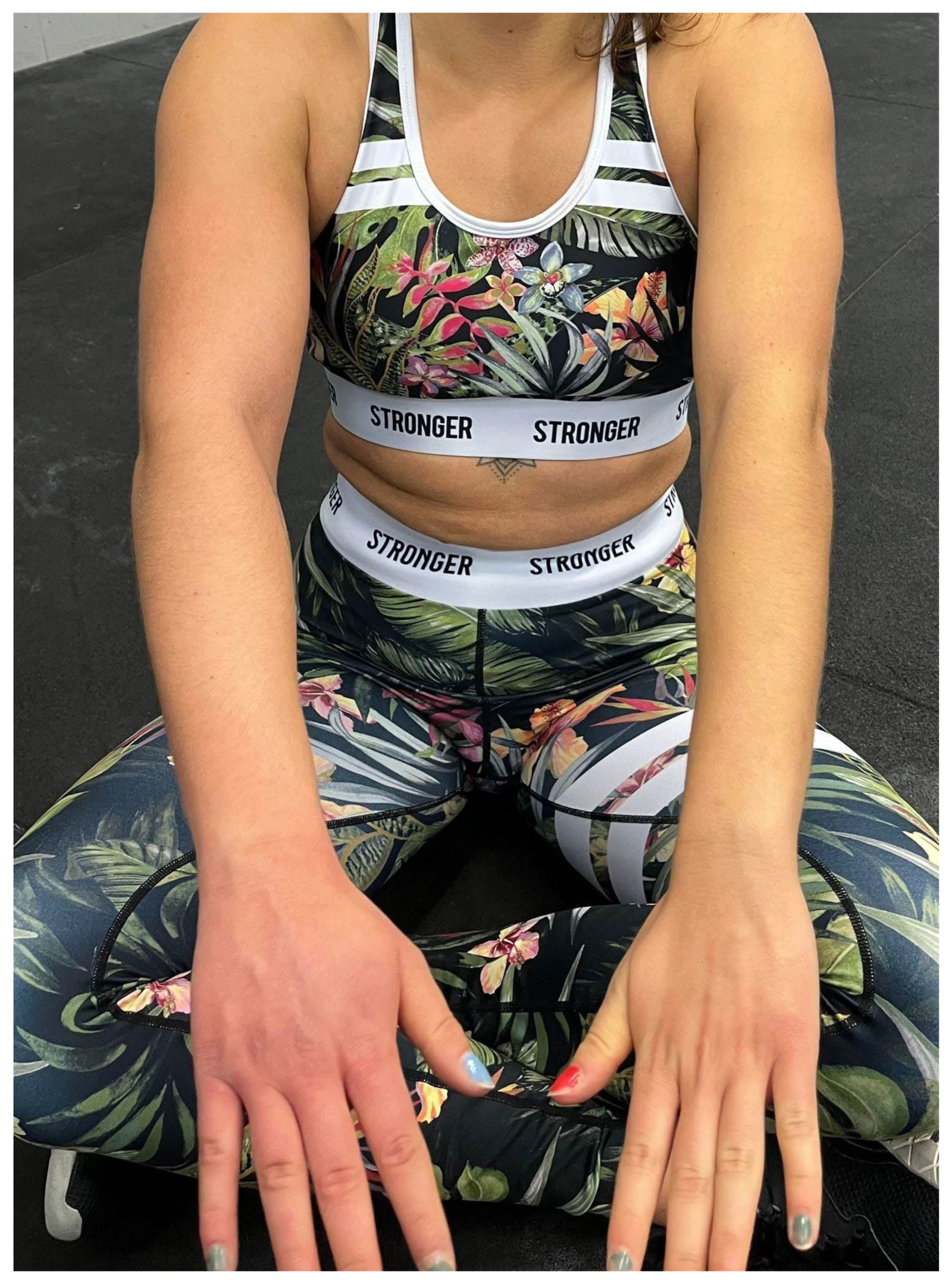
Clinical photograph showing unilateral right arm swelling and discoloration directly after intense CrossFit training. Written informed consent for publication of the image was obtained from the patient.

The patient first sought care at an emergency department because of arm swelling and pain. She was triaged by a nurse, who consulted both the on-call internal medicine physician and orthopedic surgeon. Following this assessment, the patient was discharged without direct physician evaluation, as the presenting symptoms were interpreted as being of musculoskeletal origin and related to recent physical activity. Because symptoms persisted, she presented to primary care the following day. Examination demonstrated unilateral upper extremity swelling, mild cyanotic discoloration, venous congestion, and heaviness (Figure 1). Given the unilateral exertion-associated presentation, UEDVT and PSS were suspected, and she was urgently referred back to the emergency department.

Despite this referral concern, the symptoms were again interpreted as exercise-related musculoskeletal pain or muscle strain related to recent CrossFit activity. Vascular imaging was not performed, and she was discharged without anticoagulation or further diagnostic work-up. No laboratory investigations, including D-dimer testing, were performed during either of the first two emergency department visits.

This decision was again based on the assessment that the presenting symptoms were of musculoskeletal origin and associated with recent physical activity. Over the next 24 h, symptoms persisted.

The following day, she returned to primary care because of ongoing symptoms after repeated healthcare contacts. Reassessment confirmed persistent unilateral swelling and venous congestion. She was referred once more to the emergency department with a specific recommendation for urgent duplex ultrasonography. Formal duplex ultrasonography performed and interpreted by a radiologist demonstrated deep venous thrombosis of the right axillary vein with thrombotic extension into the mid-portion of the subclavian vein, while flow in the internal jugular vein and at the jugulo-subclavian confluence remained patent, consistent with PSS. Initial laboratory investigations revealed a hemoglobin level of 129 g/L, leukocyte count of 8.9 × 10^9^/L, creatinine of 56 μmol/L, international normalized ratio (INR) of 1.1, activated partial thromboplastin time (aPTT) of 25 s, and an elevated D-dimer level of 1.6 mg/L FEU. Vital signs were unremarkable, including an oxygen saturation of 100% on room air, and there were no clinical findings suggestive of pulmonary embolism; therefore, no dedicated pulmonary imaging was performed.

The case was considered most likely related to strenuous upper extremity activity. Anticoagulation with rivaroxaban was initiated at a dose of 15 mg twice daily, followed by standard maintenance therapy, for a total treatment duration of six months. Anticoagulation resulted in a gradual improvement of acute symptoms. A comprehensive thrombophilia evaluation, including assessment of protein C, protein S, antithrombin, activated protein C resistance, prothrombin gene mutation, lupus anticoagulant, and anticardiolipin antibodies, revealed no thrombophilic abnormalities.

During follow-up, however, she continued to report residual heaviness, discomfort, visible venous collaterals, and functional limitation of the affected arm, consistent with post-thrombotic sequelae. Follow-up duplex ultrasonography performed four months after diagnosis demonstrated complete recanalization of the previously thrombosed venous segment. Despite radiological resolution, the patient continued to experience residual symptoms, including heaviness, intermittent swelling, cyanotic discoloration, impaired fine motor function, paresthesias, visible collateral veins over the shoulder and anterior chest wall, and functional limitation of the affected arm, consistent with post-thrombotic sequelae.

Given the persistence of symptoms, further evaluation for thoracic outlet pathology was undertaken approximately 18 months after the initial event. Clinical provocative testing did not demonstrate evidence of neurogenic thoracic outlet syndrome. Computed tomography angiography performed with the right arm in abduction demonstrated no evidence of arterial thoracic outlet syndrome, cervical rib, or other skeletal abnormality. Subsequent provocation venography demonstrated post-thrombotic changes with extensive collateral venous drainage but no surgically remediable dynamic venous compression. Consequently, conservative management was continued. At long-term follow-up, more than two years after the initial thrombotic event, the patient continued to report residual functional impairment and post-thrombotic symptoms. A validated upper-extremity post-thrombotic syndrome assessment score was not identified in the available medical record documentation; therefore, the presence and severity of post-thrombotic syndrome were not formally graded using a standardized scoring system in this report.

## 4. Results

### 4.1. Selection of Sources of Evidence

**Figure 2:**
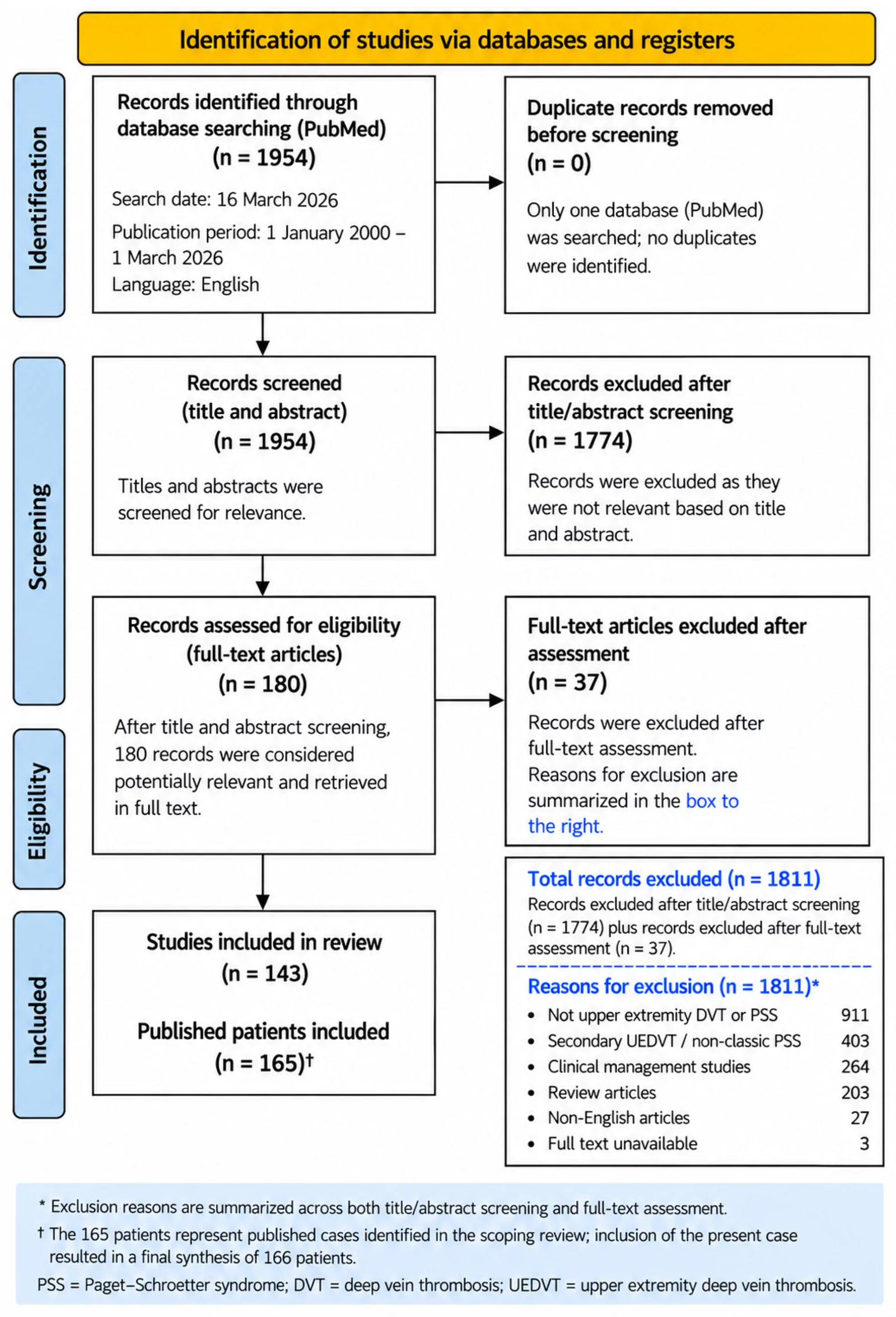
Flow diagram of record screening and study inclusion.

A total of 1954 records were identified through PubMed screening. After application of the predefined eligibility criteria, 143 studies met the inclusion criteria and contributed 165 published patients [2,7,8,9,10,11,12,13,14,15,16,17,18,19,20,21,22,23,24,25,26,27,28,29,30,31,32,33,34,35,36,37,38,39,40,41,42,43,44,45,46,47,48,49,50,51,52,53,54,55,56,57,58,59,60,61,62,63,64,65,66,67,68,69,70,71,72,73,74,75,76,77,78,79,80,81,82,83,84,85,86,87,88,89,90,91,92,93,94,95,96,97,98,99,100,101,102,103,104,105,106,107,108,109,110,111,112,113,114,115,116,117,118,119,120,121,122,123,124,125,126,127,128,129,130,131,132,133,134,135,136,137,138,139,140,141,142,143,144,145,146,147,148]. Together with the present case, the final synthesis included 166 patients. The study selection process is summarized in Figure 2.

Most included publications contributed to a single eligible patient, whereas seven studies contributed to two or more eligible cases. A complete list of included studies and their relative case contribution is provided in Supplementary Table S2.

### 4.2. Patient Characteristics, Provoking Factors, and Family History

The median patient age was 28 years (IQR, 22–38 years), and 73.5% of patients were male. Repetitive upper extremity exertion was the predominant provoking factor and was reported in 71.7% of patients. Weightlifting or gym-related activities accounted for 34.3% of cases. Thrombophilia or other hypercoagulable states were uncommon. Among the 79 patients for whom family history information was available, seven (8.9%) had a positive family history of thrombosis or hypercoagulability. Demographic characteristics, provoking activities, thrombophilia status, and family history are summarized in Table 1 and Table 2.

### 4.3. Clinical Presentation

Local upper extremity symptoms predominated. Arm swelling was reported in 95.2% of patients and arm pain in 80.1%. Cyanosis or discoloration and venous distension were each reported in approximately 70% of patients. Heaviness and functional limitation were also frequently reported. Respiratory symptoms were less common and were reported separately from pulmonary embolism, which was classified as a complication or outcome. Presenting symptoms and clinical signs are summarized in Table 3.

### 4.4. Initial Misdiagnosis and Diagnostic Delay

Initial misdiagnosis was reported in 36 patients (21.7%), while no explicit initial misdiagnosis was reported in 130 patients (78.3%). Among patients with reported initial misdiagnosis, musculoskeletal injury or muscle strain was the most common alternative diagnosis (24/36, 66.7%), followed by infectious diagnoses or cellulitis (5/36, 13.9%). Other or unspecified initial diagnoses accounted for seven cases (19.4%).

Diagnostic delay, defined as a delay occurring after the patient’s initial healthcare contact rather than patient delay from symptom onset, was reported in 45 patients (27.3% of cases with available data). Among patients with reported diagnostic delay, the duration of delay was available in 36 cases (80.0%), with a median delay of 12 days (IQR, 6–29 days) and a range of 1–365 days. Initial misdiagnosis and diagnostic delay were analyzed as distinct variables. All 36 patients with an explicitly reported initial misdiagnosis also had diagnostic delay, while an additional nine patients experienced diagnostic delay without an explicitly reported alternative initial diagnosis. Initial misdiagnosis, diagnostic delay, and diagnostic delay duration are summarized in Table 4.

### 4.5. Complications and Outcomes

Pulmonary embolism was reported in 41 patients (24.7%), whereas 125 patients (75.3%) had no reported evidence of pulmonary embolism. This reported frequency should be interpreted with caution, as it may be influenced by publication bias, selective imaging practices, and preferential reporting of more severe clinical presentations, and does not represent a population-level risk estimate. Information regarding post-thrombotic syndrome was available for only 17 patients, of whom five were reported to have developed post-thrombotic syndrome (5/17). Recurrence data were available for 156 patients, of whom 11 (7.1%) experienced recurrent thrombosis. Complications and outcomes are summarized in Table 5.

### 4.6. Treatment of Effort-Induced Upper Extremity Deep Vein Thrombosis

Treatment data were descriptively summarized across the 166 included patients. Anticoagulation was documented in 164 patients (98.8%), thrombolysis in 87 (52.4%), and surgical decompression in 71 (42.8%). Anticoagulation status was not reported for two patients. Treatment modalities were not mutually exclusive; 101 patients (60.8%) had two or more documented treatment modalities. Treatment patterns were summarized descriptively and were not analyzed comparatively. The distribution of reported treatment strategies is summarized in Table 6.

## 5. Discussion

This case report and scoping review identified a consistent early clinical pattern in reported cases of classic PSS. Patients were usually young and physically active, symptoms commonly followed repetitive or strenuous upper extremity activity, and the dominant presentation was unilateral arm swelling with pain, venous congestion, discoloration, or heaviness. Despite this recognizable constellation, diagnostic delay and initial misdiagnosis were identified as a recurring finding across the published literature.

The first major finding is the mismatch between perceived thrombotic risk and actual disease presentation. PSS often occurs in patients who do not fit the usual clinical profile of venous thromboembolism [11,13,31,48,113,114]. In the present synthesis, the median age was 28 years, most patients were male, and exertional upper-limb activity was a common precipitating factor. Furthermore, family history information was available for fewer than half of the included patients. Among those with reported data, only 8.9% had a positive family history of thrombosis or hypercoagulability. Although conclusions regarding inherited thrombotic predisposition are limited by incomplete reporting, this clinical profile may nevertheless lead clinicians to prioritize diagnoses such as sports injury, muscle strain, tendon pathology, or overuse syndromes, particularly when pain following exercise is the presenting symptom.

The second major finding was the frequent overlap between Paget–Schrotter syndrome and common musculoskeletal conditions [26,55,131]. Symptoms including arm pain, heaviness, swelling, and exercise-associated discomfort often resembled muscle strain, tendon injury, or overuse syndromes, likely contributing to the frequent occurrence of initial musculoskeletal misdiagnosis reported across the literature [24,27,49,50,58,69,74,77,80,83,86,93,102,107,138,142,143,147]. However, unilateral swelling accompanied by venous congestion, cyanotic discoloration, and exertional symptom onset was identified as a highly consistent clinical presentation among reported cases [16,44,109,112].

Third, diagnostic delay was a recurring finding across the reviewed literature [18,24,44,69,115,138,142,143]. Diagnostic delay was reported in approximately one-quarter of patients with available data. Initial misdiagnosis was reported in approximately one-fifth of cases with available data and most commonly involved musculoskeletal injury or overuse-related conditions. Several reports described prolonged diagnostic delays extending months or even years before definitive diagnosis [49,87]. These findings suggest that Paget–Schrotter syndrome may remain under-recognized during early healthcare encounters, particularly in otherwise healthy and physically active individuals. Visible collateral veins may indicate a degree of chronicity or acute-on-chronic venous obstruction, as collateral venous pathways generally require time to develop [67]. The frequent observation of venous distension and visible collateral veins in the reviewed cases may therefore partly reflect a period of unrecognized venous obstruction before diagnosis and could partly contribute to delayed recognition of the condition [77]. However, the true frequency of delayed recognition is likely underestimated, as many reports provided limited information regarding prior healthcare contacts, early diagnostic considerations, or symptom duration before definitive diagnosis.

The fourth finding was that treatment was predominantly based on anticoagulation, which was administered in nearly all reported patients, while approximately half underwent thrombolysis and more than two-fifths underwent surgical decompression. The relatively frequent use of thrombolytic therapy and thoracic outlet decompression likely reflects the unique pathophysiology of Paget–Schrotter syndrome, in which repetitive mechanical compression may contribute to both thrombus formation and persistent venous obstruction. This treatment pattern is consistent with contemporary management strategies that extend beyond anticoagulation alone and often aim to restore venous patency while addressing underlying thoracic outlet compression when present [68]. Delayed diagnosis was frequently reported across the reviewed cases and has been associated with chronic venous changes, including thrombus organization, collateral vessel formation, and persistent venous obstruction [67,77]. These factors may contribute to a greater symptom burden and influence treatment decisions. Invasive interventions, including catheter-directed thrombolysis, angioplasty, and thoracic outlet decompression, were frequently employed to restore venous patency and address thoracic outlet compression when clinically or anatomically demonstrated [68,77]. Nevertheless, considerable heterogeneity in treatment strategies was observed across reports, reflecting ongoing variation in clinical practice and the absence of universally accepted management protocols [2,68,72,77]. Because treatment data were extracted for descriptive purposes only, these findings should not be interpreted as comparative assessments of treatment efficacy.

**Figure 3:**
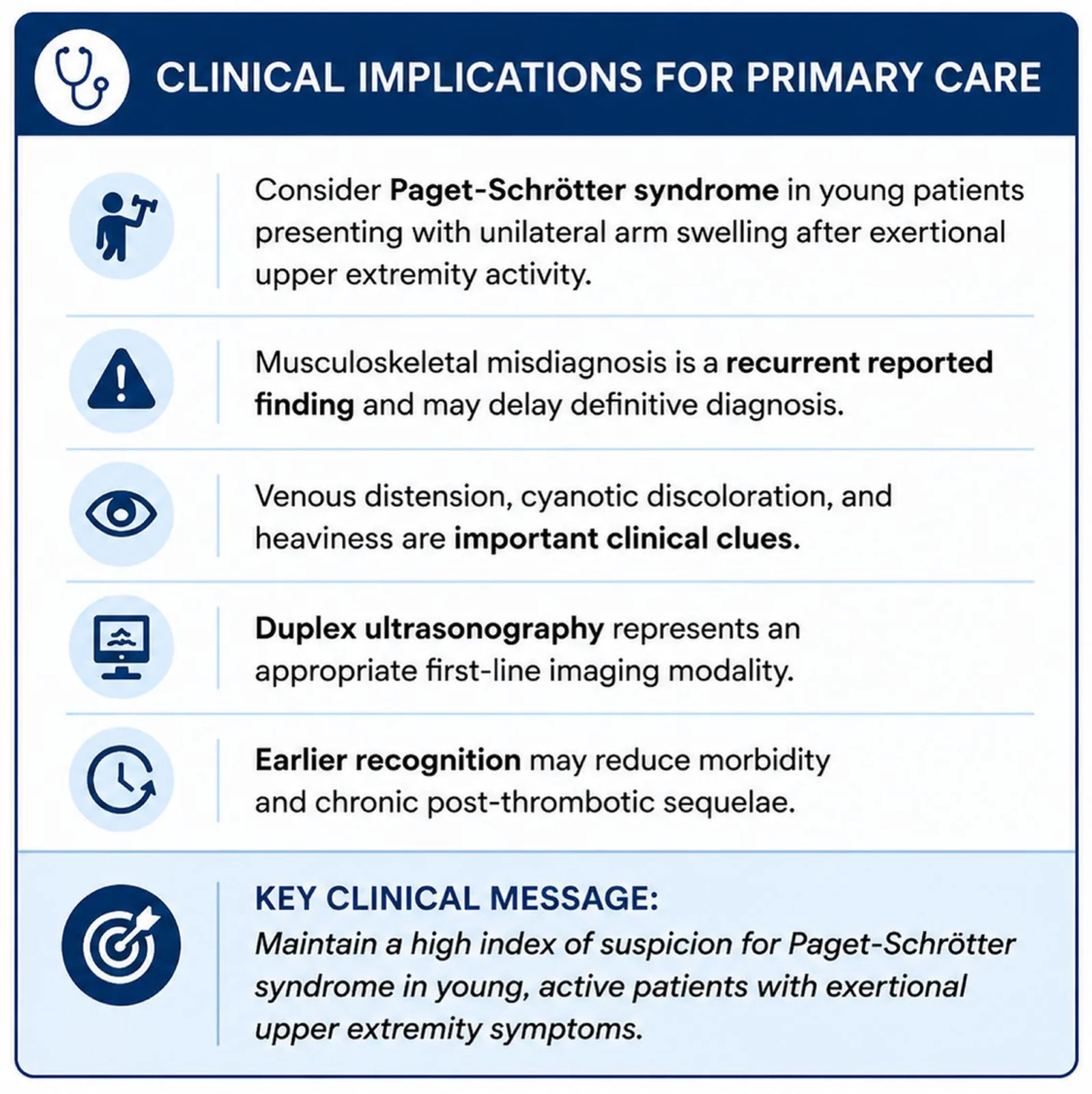
Clinical implications for primary care. The figure summarizes key clinical features that may support earlier recognition of Paget–Schrotter syndrome in low-risk patients with exertion-associated unilateral upper extremity symptoms.

Pulmonary embolism was reported in 41 published patients (24.7%). This finding should not be interpreted as a population-level risk estimate. The included evidence consisted mainly of case reports and small case series, which are prone to publication bias and may preferentially include severe, unusual, or complicated presentations. Differences in imaging practices and reporting of asymptomatic emboli may also influence this figure. Nevertheless, these reported cases highlight that PSS may be associated with clinically significant thromboembolic complications. From a primary care perspective, the practical implication is straightforward (Figure 3): young age and recent exercise should not exclude venous thrombosis when unilateral swelling is present. In patients with acute or subacute unilateral arm swelling after strenuous or repetitive upper extremity activity, especially when accompanied by venous distension, discoloration, heaviness, or functional limitation, duplex ultrasonography is an appropriate first-line investigation [112]. If ultrasound is inconclusive and clinical suspicion remains high, further vascular imaging may be required.

This review intentionally focused on classic effort-induced PSS involving the axillary-subclavian venous segment. Secondary UEDVT, catheter-associated thrombosis, malignancy-associated thrombosis, pseudo-PSS, and isolated distal upper extremity thrombosis were excluded to maintain a clinically coherent population. This approach strengthens the relevance of the findings for recognition of classic PSS, but it also limits generalizability to other forms of UEDVT.

The study has limitations. The evidence base was dominated by case reports and case series, which introduces publication bias and limits inference about incidence, risk, and prognosis. Reporting of diagnostic timelines, initial healthcare encounters, and early diagnostic impressions was inconsistent. Outcome reporting was also variable, particularly for post-thrombotic syndrome and recurrence. In addition, diagnostic delay in PSS may not be a universal phenomenon and is likely influenced by the level of knowledge and awareness among both patients and healthcare practitioners. Accordingly, our findings should not be interpreted as patient-level measures of diagnostic performance in PSS, but rather as hypothesis-generating observations highlighting recurrent diagnostic pitfalls across multiple published case reports and case series. The search was limited to English-language publications indexed in PubMed. PubMed was selected because of the focused clinical scope of the review and its emphasis on identifying case reports and case series containing patient-level information on the clinical presentation and diagnostic pathway of classic PSS. Nevertheless, the use of a single bibliographic database remains a limitation, and additional searches in databases such as Embase or Scopus may have identified further relevant reports. Consequently, the review should not be considered exhaustive, and relevant cases indexed exclusively in other databases or published in other languages may have been missed. The restriction to English-language publications may also have introduced language and geographical bias. Because diagnostic pathways, access to vascular imaging, and referral patterns may differ across healthcare systems, the observed frequencies and duration of diagnostic delay and initial misdiagnosis may not be fully representative of cases reported in non-English-language settings. In addition, the search strategy relied on free-text terminology without controlled vocabulary terms, which may have resulted in relevant reports being missed because of differences in terminology or indexing.

Furthermore, reference lists were not systematically searched, which may have reduced the completeness of case identification. Finally, although the review was reported according to PRISMA-ScR, the protocol was not registered prospectively.

Despite these limitations, the review provides a focused synthesis of early presentation and diagnostic recognition in PSS. The findings support a simple clinical message: unilateral upper extremity swelling after exertion should trigger consideration of axillary-subclavian vein thrombosis, even when the patient is young, healthy, and physically active.

## 6. Conclusions

Paget–Schrotter syndrome is an uncommon but important cause of upper extremity deep vein thrombosis in young and otherwise healthy patients. Across the published case literature, the syndrome most often presents with unilateral arm swelling, pain, discoloration, venous distension, and heaviness after strenuous or repetitive upper extremity activity. Diagnostic delay and initial musculoskeletal misdiagnosis were recurrent findings across the published case literature. Primary care and emergency clinicians should consider UEDVT when exertion-related arm symptoms are accompanied by unilateral swelling or venous congestion. Earlier recognition and timely duplex ultrasonography may help reduce delayed diagnosis and chronic post-thrombotic morbidity.

## Figures and Tables

**Table 1:** Patient demographics, provoking activities, predisposing risk factors.

Characteristic	Reported Frequency (% of Patients) *
Demographics	
Male sex	122 (73.5)
Female sex	44 (26.5)
Sex not reported	0 (0)
Age, median (IQR)	28 (22–38) years
Age not reported	5 (3.0)
Provoking activities	
Weightlifting/gym-related effort	57 (34.3)
Sports-related activity	53 (31.9)
Repetitive overhead/upper extremity activity	119 (71.7)
No provoking activity reported	0 (0)
Predisposing risk factors	
Thrombophilia	8 (4.8)

* N = 166 patients. Risk categories are not mutually exclusive. Median age was calculated from patients with reported age data.

**Table 2:** Family history.

Family History of Thrombosis/Hypercoagulability	Reported Frequency (% of Available Data) *
Positive	7 (8.9)
Negative	72 (91.1)
Family history not reported	87 (-)

* Percentages were calculated using only cases in which family history status was reported (n = 79).

**Table 3:** Symptoms and clinical signs at presentation.

Symptom/Sign	Reported Frequency (% of Patients) *
Local upper extremity symptoms/signs	
Arm swelling	158 (95.2)
Arm pain	133 (80.1)
Cyanosis/discoloration	116 (69.9)
Venous distension	117 (70.5)
Heaviness	99 (59.6)
Functional limitation	87 (52.4)
Neurological symptoms	
Paresthesia/numbness	23 (13.9)
Neck/shoulder pain	8 (4.8)
Respiratory/chest symptoms	
Dyspnea	13 (7.8)
Chest pain	7 (4.2)
Presenting symptoms/signs not reported	0 (0)

* N = 166 patients. Pulmonary embolism was treated as a complication/outcome rather than as a presenting symptom.

**Table 4:** Initial Misdiagnosis and Diagnostic Delay.

Initial Misdiagnosis and Diagnostic Delay	Reported Frequency (% of Available Data) *
Initial misdiagnosis	
Yes	36 (21.7)
No	130 (78.3)
Initial misdiagnosis not reported	0 (-)
Type of initial misdiagnosis	
Musculoskeletal injury/muscle strain	24 (66.7)
Infectious diagnosis/cellulitis	5 (13.9)
Other or unspecified initial misdiagnosis	7 (19.4)
Diagnostic delay †	
Present	45 (27.3)
Absent	120 (72.7)
Diagnostic delay not reported	1 (-)
Diagnostic delay duration	
Duration reported	36 (80.0)
Duration not reported	9 (20.0)
Diagnostic delay duration, median (IQR), days	12 (6–29)
Diagnostic delay duration range, days	1–365

* Percentages were calculated using only cases in which the respective variable was reported. † Diagnostic delay was defined as a delay occurring after the patient’s initial healthcare contact and was distinguished from patient delay (time from symptom onset to first healthcare presentation). For diagnostic delay duration, percentages were calculated among patients with reported diagnostic delay (n = 45). Initial misdiagnosis and diagnostic delay were assessed as distinct variables; diagnostic delay could be present without an explicitly reported alternative initial diagnosis.

**Table 5:** Complications and outcomes.

Complications and Outcomes	Reported Frequency (% of Available Data) *
Pulmonary embolism	
Yes	41 (24.7)
No	125 (75.3)
Pulmonary embolism not reported	0 (-)
Post-thrombotic syndrome †	
Yes	5 (5/17)
No	12 (12/17)
Post-thrombotic syndrome not reported	149 (-)
Recurrence	
Yes	11 (7.1)
No	145 (92.9)
Recurrence not reported	10 (-)

* Percentages were calculated using only cases in which the respective variable was reported. † Post-thrombotic syndrome is presented as raw fractions because outcome data were available for only 17 patients.

**Table 6:** Treatment of effort-induced upper extremity deep vein thrombosis.

Treatment	Frequency/Percentage of Patients *	Not Reported, n
Anticoagulation	164 (98.8)	2
Thrombolysis	87 (52.4)	0
Surgical decompression	71 (42.8)	0

* N = 166 patients. Treatment categories are not mutually exclusive. A total of 101 patients (60.8%) had two or more documented treatment modalities.

## Data Availability

The data supporting the findings of this study are contained within the article and its Supplementary Materials. The extracted dataset used for the scoping review is available from the corresponding author upon reasonable request. Patient-level data from the presented case report are not publicly available in order to protect patient privacy.

## Supplementary Materials

The following supporting information can be downloaded at: https://www.mdpi.com/article/10.3390/clinpract16090172/s1, Table S1: PRISMA-ScR Checklist [6]; Table S2: Complete list of included studies with hyperlinks and contributed cases.

## References

[1] Schwenke M., Goldman R.E., Sarkeshik A.A., King E.C. (2022). Subclavian Effort Thrombosis: Pathophysiology, Diagnosis, and Management. Semin. Interv. Radiol..

[2] Ibrahim R., Dashkova I., Williams M., Kozikowski A., Abrol N., Gandhi A., Pekmezaris R. (2017). Paget-Schroetter Syndrome in the Absence of Common Predisposing Factors: A Case Report. Thromb. J..

[3] Hangge P., Rotellini-Coltvet L., Deipolyi A.R., Albadawi H., Oklu R. (2017). Paget-Schroetter Syndrome: Treatment of Venous Thrombosis and Outcomes. Cardiovasc. Diagn. Ther..

[4] Illig K.A., Doyle A.J. (2010). A Comprehensive Review of Paget-Schroetter Syndrome. J. Vasc. Surg..

[5] Arksey H., O’Malley L. (2005). Scoping Studies: Towards a Methodological Framework. Int. J. Soc. Res. Methodol..

[6] Tricco A.C., Lillie E., Zarin W., O’Brien K.K., Colquhoun H., Levac D., Moher D., Peters M.D.J., Horsley T., Weeks L. (2018). PRISMA Extension for Scoping Reviews (PRISMAScR): Checklist and Explanation. Ann. Intern. Med..

[7] Uemura A., Osaka I., Fujimoto H. (2000). Acute Axillosubclavian Vein Thrombosis (Paget–Schroetter Syndrome) Detected by TC-99M MAA during Pulmonary Perfusion Scintigraphy. Clin. Nucl. Med..

[8] Sullivan V.V., Wolk S.W., Whitehouse W.M. (2001). Simultaneous Bilateral Upper Extremity Venous Thrombosis in a Factor V Leiden Heterozygote. Vasc. Surg..

[9] Taira N., Mano M., Asano H., Takashima S., Nishi H., Fukuda K., Komatsubara S. (2001). Primary Subclavian Venous Thrombosis Which Developed After Sleeping with the Arm in an Outstretched Position: Report of a Case. Surg. Today.

[10] Isaka N., Yamada N., Araki S., Onishi K., Motoyasu M., Okinaka T., Ito M., Nakano T. (2001). Multiple Pulmonary Emboli with Pulmonary Hypertension Caused by Effort Thrombosis and Effective Balloon Venoplasty of the Subclavian Vein. Jpn. Circ. J..

[11] Zell L., Kindermann W., Marschall F., Scheffler P., Gross J., Buchter A. (2001). Paget-Schroetter Syndrome in Sports Activities. Angiology.

[12] Filis K.A., Nguyen T.Q., Olcott C. (2002). Subclavian Vein Thrombosis Caused by an Unusual Congenital Clavicular Anomaly in an Atypical Anatomic Position. J. Vasc. Surg..

[13] DiFelice G.S., Paletta G.A., Phillips B.B., Wright R.W. (2002). Effort Thrombosis in the Elite Throwing Athlete. Am. J. Sports Med..

[14] Pedrosa I., Aschkenasi C., Hamdan A., Rofsky N.M. (2002). Effort-Induced Thrombosis: Diagnosis with Three-Dimensional MR Venography. Emerg. Radiol..

[15] Heis H.A., Bani-Hani K.E. Effort Thrombosis of the Subclavian-Axillary Vein. https://pubmed.ncbi.nlm.nih.gov/12436122/.

[16] Sevestre M., Kalka C., Irwin W.T., Ahari H.K., Schainfeld R.M. (2003). Paget-Schroëtter Syndrome: What to Do?. Catheter. Cardiovasc. Interv..

[17] McGlinchey P.G., Shamsuddin S.A., Kidney J.C. Effort-Induced Thrombosis of the Subclavian Vein—A Case of Paget-Schroetter Syndrome. https://pmc.ncbi.nlm.nih.gov/articles/PMC2475444/.

[18] Vijaysadan V., Zimmerman A.M., Pajaro R.E. (2005). Paget-Schroetter Syndrome in the Young and Active. J. Am. Board Fam. Med..

[19] Abbasi A.A. (2005). An Unusual Swelling in the Neck. Emerg. Med. J..

[20] Keisler B.D., Armsey T.D. (2005). Paget-Schroetter Syndrome in an Overhead Athlete. Curr. Sports Med. Rep..

[21] Fiorentini C., Mattioli S., Graziosi F., Bonfiglioli R., Armstrong T.J., Violante F.S. (2005). Occupational Relevance of Subclavian Vein Thrombosis in Association with Thoracic Outlet Syndrome. Scand. J. Work Environ. Health.

[22] Özçakar L., Kaymak B., Turan S., Akal M., Enön S., Okuyan H. (2006). Thoracic Outlet Syndrome, Paget–Schroetter Syndrome and Aberrant Subclavian Artery in a Young Man. Jt. Bone Spine.

[23] Meier M.A., Rubenfire M. (2006). Life-Threatening Acute and Chronic Thromboembolic Pulmonary Hypertension and Subclavian Vein Thrombosis. Clin. Cardiol..

[24] Hurley W.L., Comins S.A., Green R.M., Canizzaro J. Atraumatic Subclavian Vein Thrombosis in a Collegiate Baseball Player: A Case Report. https://pmc.ncbi.nlm.nih.gov/articles/PMC1472647/.

[25] Liang H.-W., Su T.-C., Hwang B.-S., Hung M.-H. (2006). Effort Thrombosis of the Upper Extremities Related to an Arm Stretching Exercise. J. Formos. Med. Assoc..

[26] Hendrickson C.D., Godek A., Schmidt P. (2005). Paget-Schroetter Syndrome in a Collegiate Football Player. Clin. J. Sport Med..

[27] Hegedus E.J., Cooper L., Cook C. (2006). Diagnosis of a Rare Source of Upper Extremity Symptoms in a Healthy Female after Weight Lifting. J. Orthop. Sports Phys. Ther..

[28] Youssef M.Y.Z., Taweel T.A., Asfar S., Abdella N. (2006). Effort-Induced Venous Thrombosis of the Upper Limbs. Med. Princ. Pract..

[29] Toya N., Fujita T., Ohki T. (2007). Push-Up Exercise Induced Thrombosis of the Subclavian Vein in a Young Woman: Report of a Case. Surg. Today.

[30] Dintaman J., Watson C., Fox C.J., Hoover N., Roberts S., Gillespie D.L. (2006). Case of Adolescent with Paget-Schroetter Syndrome and Underlying Thrombophilia Due to an Elevated Lipoprotein (A). Pediatr. Blood Cancer.

[31] Goel S., Clair D.G., Carman T.L. (2007). An 18-Year-Old with Effort-Related Arm Swelling. Clevel. Clin. J. Med..

[32] Gursel O.L., Emin E.G. Paget-Schroetter Syndrome. https://pubmed.ncbi.nlm.nih.gov/17548917/.

[33] Roche-Nagle G., Ryan R., Barry M., Brophy D. (2007). Effort Thrombosis of the Upper Extremity in a Young Sportsman: Paget–Schroetter Syndrome. Br. J. Sports Med..

[34] Colak M.C., Kocatürk H., Bayram E. (2008). Paget-Schroetter Syndrome/Paget-Schroetter Sendromu. Anadolu Kardiyol. Derg..

[35] Wallis A., Winterborn R., Jones L. (2008). A Simple Case of Upper Limb Venous Thrombosis Requiring Anticoagulation?. Clin. Med..

[36] Phipps C., Ng H.J. (2008). Upper Limb Deep Vein Thrombosis and Portable Computer Games. Am. J. Med..

[37] Snead D., Marberry K.M., Rowdon G. (2009). Unique Treatment Regimen for Effort Thrombosis in the Nondominant Extremity of an Overhead Athlete: A Case Report. J. Athl. Train..

[38] Bushnell B.D., Anz A.W., Dugger K., Sakryd G.A., Noonan T.J. (2009). Effort Thrombosis Presenting as Pulmonary Embolism in a Professional Baseball Pitcher. Sports Health A Multidiscip. Approach.

[39] Ilhan E. (2009). Subclavian Vein Thrombosis Extending into the Internal Jugular Vein: Paget-von Schroetter Syndrome. J. Clin. Med. Res..

[40] Girma F. (2010). Upperr Extremity Deep Vein Thrombosis in a 25 Years Old Apparently Healthy Man. Pan Afr. Med. J..

[41] Seeger M., Bewig B. (2010). Paget–Schroetter Syndrome. N. Engl. J. Med..

[42] Özçakar L., Dönmez G., Yörübulut M., Aydoğ S.T., Demirel H., Paşaoğlu İ., Doral M.N. (2009). Paget-Schroetter Syndrome Forerunning the Diagnoses of Thoracic Outlet Syndrome and Thrombophilia. Clin. Appl. Thromb./Hemost..

[43] Dhillon R.K., Spahr C.D. (2010). Two Cases of Upper-Extremity Swelling. Pediatr. Emerg. Care.

[44] Hobeika C., Meziane M.A., Sands M.J., Lababede O. (2010). Paget-Schroetter Syndrome. J. Thorac. Imaging.

[45] Pysklywec M., Cina C.S. (2010). Work-related Effort Thrombosis in a Millwright: A Case Report. Am. J. Ind. Med..

[46] Allana A.M.B., Teo L.L.S., Chuah B.Y.S., Liu T.C. (2011). Cheah Effort Thrombosis in a Young Triathlete: An Unusual Presentation of Painless Neck Swelling Secondary to Right Brachiocephalic Vein Thrombosis. Singap. Med. J..

[47] Alcelik A., Savli H., Zeyrek A., Yalcin A. (2011). Hand Knitting Induced Thrombosis of the Subclavian Vein in a Young Woman: An Unusual Cause of Paget-Schoetter Syndrome. Mediterr. J. Hematol. Infect. Dis..

[48] Stainsby B.E., Muir B.J., Miners A.L. (2012). Upper Extremity Deep Vein Thrombosis Presenting to a Chiropractic Clinic: A Description of 2 Cases. J. Chiropr. Med..

[49] Rowan T.L., Kazemi M. Paget Schroetter Syndrome: A Case Study of the Chiropractor’s Role in Recognizing and Comanaging an Important Condition. https://pmc.ncbi.nlm.nih.gov/articles/PMC3501911/.

[50] Sieniewicz B.J., McCabe S. (2011). Upper Extremity Deep Venous Thrombosis….Can You Spot the Culprit?: Figure 1. Emerg. Med. J..

[51] Sayin A., Gungor H., Bilgin M., Erturk U. (2012). Paget-von Schröetter Syndrome: Upper Extremity Deep Vein Thrombosis after Heavy Exercise. Turk. Kardiyol. Dern. Ars..

[52] O’Keefe S., Carmody K.A. (2013). Paget-Schroetter Syndrome Diagnosed by Bedside Emergency Physician Performed Ultrasound: A Case Report. J. Emerg. Med..

[53] Dep A., Concannon E., Hugh S.M.M., Burke P. (2013). Paget-Schrotter Syndrome and Complications of Management. BMJ Case Rep..

[54] Coughlin L.M., Koenig K.N., Clark P.M. (2013). Claviculectomy with Thrombectomy for Management of Paget-Schroetter Syndrome in a Patient with Chronic Clavicular Malunion. Ann. Vasc. Surg..

[55] Kohen D., Hanhan S., Bellah R. (2013). Paget-Schroetter Syndrome in a Lacrosse Player. Del. Med. J..

[56] Drakos N., Gausche-Hill M. (2012). A Case Report: A Young Waiter with Paget-Schroetter Syndrome. J. Emerg. Med..

[57] Nitz A.J., Nitz J.A. VASCULAR THORACIC OUTLET IN a COMPETITIVE SWIMMER: A CASE REPORT. https://pmc.ncbi.nlm.nih.gov/articles/PMC3578436/.

[58] Kidd M., Broderick V. (2014). An Unusual Presentation of a Swollen Arm: A Case Report. J. Med. Case Rep..

[59] Kellar J., Trigger C. (2014). Thoracic Outlet Syndrome with Secondary Paget Schröetter Syndrome: A Rare Case of Effort-Induced Thrombosis of the Upper Extremity. West. J. Emerg. Med..

[60] Young K., Tunstall O., Mumford A. (2014). Subclavian Vein Thrombosis in an Otherwise Healthy 9-Year-Old Boy. BMJ Case Rep..

[61] Jabri H., Mukherjee S., Sanghavi D., Chalise S. (2014). Bilateral Upper Extremity DVT in a 43-Year-Old Man: Is It Thoracic Outlet Syndrome?!. Case Rep. Med..

[62] Waterman G. (2014). Effort Thrombosis (Paget-Schroetter Syndrome) in a 16-Year-Old Male. Am. J. Case Rep..

[63] Meena M., Harish S., Kewlani J.P., Gupta N., Meena V.K. (2015). Paget-Schroetter Syndrome. Chin. Med. J..

[64] Keene D.J. (2014). Upper Extremity Deep Vein Thrombosis (Paget-Schroetter Syndrome) after Surfing: A Case Report. Man. Ther..

[65] Lutter C., Monasterio E., Schöffl V. (2015). Rock Climbing-Related Subclavian Vein Thrombosis. BMJ Case Rep..

[66] Thiruchelvam N., Mbuvah F., Kistangari G., Anumandla A.K.R. (2015). Upper-Limb Deep Vein Thrombosis in Paget-Schroetter Syndrome. Clevel. Clin. J. Med..

[67] Shimada T., Tounai T., Syoji T., Fukumoto Y. (2015). Acute Pulmonary Embolism Due to Paget-Schroetter Syndrome. Intern. Med..

[68] Stein C.M., McLeod A., Devine L.A. (2014). Spontaneous Deep Vein Thrombosis in the Upper Extremity of a 45-Year-Old Woman. Can. Med. Assoc. J..

[69] Aytekin E., Dogan Y.P., Okur S.C., Burnaz O., Caglar N.S. (2015). Differential Diagnosis of a Rare Case of Upper Limb Pain: Paget-Schroetter Syndrome in a Doner Kebab Chef. J. Phys. Ther. Sci..

[70] Shennib H., Hickle K., Bowles B. (2015). Axillary Vein Thrombosis Induced by an Increasingly Popular Oscillating Dumbbell Exercise Device: A Case Report. J. Cardiothorac. Surg..

[71] Beasley R., Braithwaite I., Evans R. (2015). Upper Extremity Deep Vein Thrombosis in a TV Cameraman. Occup. Med..

[72] Fleta G.E., Blanco Á.T., Palonés F.G., Monzón E.O. (2016). Combined Non-Surgical Treatment for Paget–Schröetter Syndrome: A Case Report. J. Med. Case Rep..

[73] Fundora M.P., Rudnick C., Barbur C. (2015). Spontaneous Upper Extremity Venous Thrombosis in a Collegiate Soccer Player. Pediatr. Emerg. Care.

[74] Ijaopo R., Oguntolu V., DCosta D., Garnham A., Hobbs S. (2016). A Case of Paget-Schroetter Syndrome (PSS) in a Young Judo Tutor: A Case Report. J. Med. Case Rep..

[75] Jourdain V., Goldenberg W.D., Matteucci M., Auten J. (2015). Paget-Schroetter Syndrome: Diagnostic Limitations of Imaging Upper Extremity Deep Vein Thrombosis. Am. J. Emerg. Med..

[76] Norinsky A.B., Espinosa J., Kianmajd M., DiLeonardo F. (2015). Painless Acrocyanosis: Paget-Schroetter Syndrome Secondary to Thoracic Outlet Obstruction from Muscle Hypertrophy. Am. J. Emerg. Med..

[77] Katana V.G., Weiss J.S. (2016). Venous Thoracic Outlet Syndrome: The Role of Early Rib Resection. Mil. Med..

[78] Stake S., Du Breuil A.L., Close J. (2016). Upper Extremity Deep Vein Thromboses: The Bowler and the Barista. Case Rep. Vasc. Med..

[79] Bullock C., Johnston A.M. (2015). Upper Extremity Deep Vein Thrombosis in a Military Patient. J. R. Army Med. Corps.

[80] VanWye W.R., Pinerola J., Ogle K.C., Wallmann H.W. SCREENING FOR REFERRAL BY a SPORTS PHYSICAL THERAPIST REVEALS AN EFFORT THROMBOSIS IN a COLLEGIATE PITCHER: A CASE REPORT. https://pmc.ncbi.nlm.nih.gov/articles/PMC4970850/.

[81] Pinar M.P., Toledo R.P., Viana L.B., Del Pozo J.S.G. (2017). Deep Vein Thrombosis in Upper Limb in a Weightlifter. Open Access Maced. J. Med. Sci..

[82] Hasegawa T., Tabata H., Kagoshima M. (2017). A Case of Upper Extremity Deep Vein Thrombosis with Long-Term Patency Using Pharmaco-Mechanical Catheter-Directed Thrombolysis in the Acute Phase. J. Cardiol. Cases.

[83] Kaczynski J., Sathiananthan J. (2017). Paget-Schroetter Syndrome Complicated by an Incidental Pulmonary Embolism. BMJ Case Rep..

[84] Lipe D.N. (2017). Young Woman with a Blue, Painful and Swollen Arm. Ann. Emerg. Med..

[85] Chu A.S., Harkness J., Witmer C.M. (2016). Spontaneous Subclavian Vein Thrombosis in a Healthy Adolescent Cheerleader. Pediatr. Emerg. Care.

[86] Ringhouse B., Jackson C. (2017). Bringing to Light Symptoms and Treatments of Effort Thrombosis (Paget–Schroetter Syndrome) in the Military Population, a Case Study. Mil. Med..

[87] Sharma H., Tiwari A. (2017). Recurrent Upper Extremity Thrombosis Associated with Overactivity: A Case of Delayed Diagnosis of Paget-Schroetter Syndrome. Case Rep. Vasc. Med..

[88] Lawless S.M., Samson R. (2017). Urschel’s Sign in Paget Schroetter Syndrome. Am. J. Med..

[89] DeLisa L.C., Hensley C.P., Jackson S. (2016). Diagnosis of Paget-Schroetter Syndrome/Primary Effort Thrombosis in a Recreational Weight Lifter. Phys. Ther..

[90] Yagi S., Mitsugi M., Sangawa T., Akaike M., Sata M. (2017). Paget-Schroetter Syndrome in a Baseball Pitcher. Int. Heart J..

[91] Noto T., Hashimoto G., Takagi T., Awaya T., Araki T., Shiba M., Iijima R., Hara H., Moroi M., Nakamura M. (2017). Paget-Schroetter Syndrome Resulting from Thoracic Outlet Syndrome and KAATSU Training. Intern. Med..

[92] Sancho-González I., Bonilla-Hernández M.V., Ibañez-Muñoz D., Vicente-Campos D., Chicharro J.L. (2016). Upper Extremity Deep Vein Thrombosis in a Triathlete: Again Intense Endurance Exercise as a Thrombogenic Risk. Am. J. Emerg. Med..

[93] Garg V., Poon G., Tan A., Poon K.B. (2018). Paget-Schroetter Syndrome as a Result of 1st Rib Stress Fracture Due to Gym Activity Presenting with Urschel’s Sign—A Case Report and Review of Literature. Int. J. Surg. Case Rep..

[94] Glavich G., Gourley J., Fong V. (2017). Paget-Schroetter Syndrome with Bilateral Pulmonary Emboli. Radiol. Case Rep..

[95] Godsey J.M., Stickles S.P. (2017). Young Man with Left Arm Pain and Swelling. Ann. Emerg. Med..

[96] Yunce M., Sharma A., Braunstein E., Streiff M.B., Lum Y.W. (2018). A Case Report on 2 Unique Presentations of Upper Extremity Deep Vein Thrombosis. Medicine.

[97] Fenando A., Mujer M., Rai M.P., Alratroot A. (2018). Paget-Schroetter Syndrome. BMJ Case Rep..

[98] Franco J., Medina F., Formiga F., Huerta J., Arbe G., Charte A. (2017). Paget–Schroetter Syndrome. QJM Int. J. Med..

[99] Alva H., Goyeneche N., Fletcher M., Warrier R. (2019). Sports Injury or Venous Thrombosis?. Clin. Pediatr..

[100] Sahashi Y., Naito J., Kawasaki M. (2019). Recurrent Pulmonary Embolism Related with Paget–Schroetter Syndrome: A Case Report. Eur. Heart J. Case Rep..

[101] Higuchi R., Miyawaki M., Yasuga Y., Tomobuchi A., Shigyo H., Nakatani K., Mitsusada N., Hiraoka H., Senzaki N., Tagami N. (2019). Paget–Schroetter Syndrome Accompanied by Pulmonary Thromboembolism: A Case Report. J. Cardiol. Cases.

[102] Lazea C., Asavoaie C. (2019). Paget–Schroetter Syndrome in a Teenager after Throwing Firecrackers—A Case Report. Niger. J. Clin. Pract..

[103] Weaver L.A., Kanter C.R., Costantino T.G. (2019). Effort Thrombosis Provoked by Saxophone Performance. J. Emerg. Med..

[104] Kubota T., Inakuma Y., Cammack I., Kisa K. (2018). A Swollen Arm after Backcountry Snowboarding in Japan. J. Travel Med..

[105] Myers L.C., Li M.D., Kalva S., Lai P.S. (2019). Pulmonary Infarction Due to Paget-Schroetter Syndrome and Nephrotic Syndrome. Am. J. Case Rep..

[106] Umana E., Elsherif M., Binchy J. (2019). A Case of Paget-Schroetter Syndrome in a Young Male after Lifting Weights. Ir. Med. J..

[107] Mattox R., Trager R.J., Kettner N.W. (2019). Effort Thrombosis in 2 Athletes Suspected of Musculoskeletal Injury. J. Chiropr. Med..

[108] Musi M.E., Melher A., Bromfield A., Rochon P.J., Davis C.B. (2020). Effort Thrombosis Presenting as Unilateral “Pumped” Arm in a Climber. Wilderness Environ. Med..

[109] Pesser N., Van Den Houten M.M.L., Van Sambeek M.R.H.M., Teijink J.A.W. (2020). Venous Thoracic Outlet Syndrome Caused by Double Compression of the Axillosubclavian Vein: A Case Report. EJVES Vasc. Forum.

[110] Osman M., Afridi F.G., Sidawy A.N., Lala S. (2020). Paget-Schroetter Syndrome in Pregnancy: A Case Report and Discussion of Management Options. J. Vasc. Surg. Cases Innov. Tech..

[111] Davis R., Reed E., Kraus C. (2020). Arm Pain and Swelling in a College Swimmer: A Case of Paget-Schroetter Syndrome. J. Osteopath. Med..

[112] El-Attrache A., Kephart E. (2020). Paget-Schroetter Syndrome: A Case Report of Diagnosis, Treatment, and Outcome in a Healthy 18-Year-Old Athletic Swimmer. Physician Sportsmed..

[113] Tanabe H., Miyamori D., Shigenobu Y., Ito Y., Kametani T., Kakimoto M., Kawahara A., Kikuchi Y., Kobayashi T., Otani Y. (2020). Two Patients with Paget-Schroetter Syndrome That Were Successfully Diagnosed by Doppler Ultrasonography: Case Studies with a Literature Review. Intern. Med..

[114] Nakagami F., Yuasa K., Ohno M., Rakugi H. (2020). A Baseball Player with a Swollen Arm. Intern. Med..

[115] Zakaria A., Gill J., Nishi L.M., Nadwodny J., Pujalte G.G.A. (2020). Soccer Player with Unusual Right Shoulder and Arm Pain and Swelling. J. Prim. Health Care.

[116] Garbrecht J.D., Reynolds W., Rosenthal M.D. (2020). Upper Extremity Effort Thrombosis. J. Orthop. Sports Phys. Ther..

[117] Ramadan S.M., Kasfiki E.V., Kelly C., Ali I. Primary Upper Extremity Deep Vein Thrombosis (Effort Thrombosis). https://pubmed.ncbi.nlm.nih.gov/34190744/.

[118] Gani A., Lucas G., Refson J.S., El-Karim A. (2021). Paget-Schroetter Syndrome in a Young Fitness Enthusiast with a Negative D-Dimer: Highlighting the Balance between Clinical Suspicion and Diagnostic Modalities. BMJ Case Rep..

[119] Adekoya O. (2021). A Case of Upper Limb Venous Thrombosis in Paget-Schroetter Syndrome. Cureus.

[120] Toros S.Z., Ertugay O.C., Celep S., Tanrikulu H. (2020). Paget Schroetter Syndrome and Chylothorax after Rhinoplasty. J. Craniofacial Surg..

[121] Maxwell M., Ebadi F., Hogan E. (2021). Delayed Diagnosis of Paget-Schroetter Syndrome in a Patient with COVID-19. Rhode Isl. Med. J..

[122] Sangani V., Pokal M., Balla M., Gayam V., Konala V.M. (2021). Paget-Schroetter Syndrome in a Young Female. J. Investig. Med. High Impact Case Rep..

[123] De Siqueira Filho J.T., De Paula R.L., Régis A.C.A., Figueiredo A.R., Von Mühlen B., Peres J.V.F. (2021). Paget-von Schroetter Syndrome. Int. J. Surg. Case Rep..

[124] Lee Y.S., Ng F.H., Pan N.Y., Fai J.K. (2021). MA Paget-Schroetter Syndrome in a Non Athlete—A Case Report. Radiol. Case Rep..

[125] Hollabaugh W.L., Bowman E.N. (2022). Effort-Associated Thrombosis: Imaging Is Key When Suspicion Is High. A Case Report. Curr. Sports Med. Rep..

[126] Endara S.A., Dávalos G.A., Fierro C.H., Montero A.R., Molina G.A. (2022). Paget-Schroetter Syndrome in an Active Young Female after Unsupervised Exercise. Int. J. Surg. Case Rep..

[127] Oliveira I., Leal F., Santos L., Pinto J.A., Nogueira L., Mesquita M. (2022). Venous Thoracic Outlet Syndrome: When Exercising May Be Discouraged. Clin. Case Rep..

[128] Suwal A., Rijal S.S., Shrestha M., Munankami S., Basnet S. (2022). Paget-Schroetter Syndrome: A Rare Case of Upper Extremity Deep Vein Thrombosis in a Young Swimmer. Cureus.

[129] Wankhade B.S., ElKhouly A.E., Alrais Z.F., Kholi M.H.I.A.E. (2022). Paget–Schroetter Syndrome. Int. J. Crit. Illn. Inj. Sci..

[130] Singh A., Kaur M., Singh A., Bhatti A., Patel R. (2022). Paget-Schroetter Syndrome (PSS) and Adjunctive Treatment with Mechanical Aspiration Thrombectomy and Catheter Directed Thrombolysis: A Case Report and Review of Literature. Cureus.

[131] Taha K., Breslín T., Moriarty J., Ali S., Louw B. (2022). Diagnosing Paget-Schroetter Syndrome Using Point of Care Ultrasound (POCUS). POCUS J..

[132] Heo J., Medani K., Juma H. (2022). Paget-Schroetter Syndrome from Manual Handling at Work: Case Report and Review of the Literature. Am. J. Ind. Med..

[133] O’Donnell M.T., Huang M.L., Gerling K.A., Rasmussen T.E., White J.M., Koelling E.E. (2022). Use of MEDEVAC Resources in Austere Settings: Paget–Schroetter in the Deployed Environment. Mil. Med..

[134] Tahirović E., Granov N. (2023). A Man in His 50s with an Unusual Network of Small Veins in the Left Infraclavicular Region—Paget-Schroetter Syndrome. JAMA Cardiol..

[135] Yang A., Seevanayagam S. (2023). Left Brachiocephalic Venous Thrombus Initially Presenting as Acute Aortic Syndrome. J. Surg. Case Rep..

[136] Pesonen L.O., Knol M.C., Kreimier E.L., Jackson M.W., Heidenreich M.J., Aziz A. (2022). Successful Paraclavicular Surgical Decompression for Recurrent Venous Thoracic Outlet Syndrome Following Transaxillary First Rib Resection in an Elite Athlete. Vascular.

[137] Pejkova S., Aleksovski D., Hadjitrifon S. (2023). Paget-Schroetter Syndrome: The Importance of Early Detection and Effective Surgical Intervention. Arch. Clin. Cases.

[138] Crossland D.T., Overturf M.D. (2024). Paget-Schroetter Syndrome: A Case of a Young Weightlifter. Cureus.

[139] Trinh K., Pessegueiro A.M. (2024). Exercise-Induced Venous Thrombosis of the Upper Extremity: A Case Report. SAGE Open Med. Case Rep..

[140] Salem S.B., Shrestha S., Ravikumar J., Huynh L., Sara S. (2024). Effort-Induced Deep Vein Thrombosis of the Right Upper Extremity. Clin. Case Rep..

[141] Ebrahim M.A., Adhikari B., Wazir H., Bhattarai H., Chalise S. (2024). Beyond an Upper Extremity Clot: A Case Report of Paget-Schroetter Syndrome. Radiol. Case Rep..

[142] Pantaleão A.N., Goudot G., Becari L., Jeunon V., Bello G.A., De Moraes A.G. (2023). Pulmonary Embolism Following an Undiagnosed Paget-Schroetter Syndrome: A Case Report and Review of the Literature. Physician Sportsmed..

[143] Lungu A.M., Andrei I.M., Uscoiu G., Grigore M., Iliesiu A.M. (2024). A Rare Cause of Deep Vein Thrombosis in a Young Orchestra Conductor. Diagnostics.

[144] Ma J., Poquiz P.H. (2025). A Case of Paget-Schroetter Syndrome in a Young, Healthy Woman. Cureus.

[145] Sato T., Ikemoto T., Tsunoda R., Koyama J. (2025). A Case of Paget-Schroetter Syndrome Successfully Treated with Endovascular Treatment. Cureus.

[146] O’Toole F., Van Ee S.K., Madison D., DiVincenzo K., Tran K., Mohamed A. (2025). Subacute Paget–Schroetter with Rapid Recurrence and Concurrent Pulmonary Emboli: A Case Report. Front. Surg..

[147] Abouafech A., Oar D.P., Chapman S.R., Singh G., Westfall S., Bhagratie J. (2025). An Unexpected Complication of Training: A Rare Case of Paget-Schroetter Syndrome in an Adolescent Athlete. Cureus.

[148] Inaba S., Okumura H., Kawashima A. (2024). Thoracic Outlet Syndrome and Thrombosis: A Case of Paget-Schroetter Syndrome Triggered by Prolonged Strap-Holding. Intern. Med..

