# Supplementary material for: Paget–Schrotter Syndrome in the Primary Care Setting: A Case Report and Scoping Review of Clinical Presentation and Diagnostic Delay
Source: Clin Pract. 2026 Sep 19;16(9):172. doi: 10.3390/clinpract16090172 (PMC13604958; doi:10.3390/clinpract16090172)
Supplement: Supplementary file 1 [file clinpract-16-00172-s001.zip › supplementary Table S1 PRISMA-SCR Checklist PSS 18092026.pdf]

Supplementary Table S1. PRISMA-ScR Checklist

SECTION	ITEM	PRISMA-ScR CHECKLIST ITEM	REPORTED ON PAGE(S)
TITLE	1	Identify the report as a scoping review.	1
ABSTRACT	2	Provide a structured summary including background, objectives, eligibility criteria, sources of evidence, charting methods, results, and conclusions.	1
INTRODUCTION	3	Describe the rationale for the review in the context of what is already known.	2
INTRODUCTION	4	Provide an explicit statement of the questions and objectives being addressed.	2
METHODS	5	Indicate whether a review protocol exists and where it can be accessed.	Not registered; p. 13
METHODS	6	Specify characteristics of the sources of evidence used as eligibility criteria.	3
METHODS	7	Describe all information sources used in the search.	3
METHODS	8	Present the full electronic search strategy.	3
METHODS	9	State the process for selecting sources of evidence.	3
METHODS	10	Describe the methods of data charting.	3-4
METHODS	11	List and define all variables for which data were sought.	3-4
METHODS	12	If done, provide a rationale for critical appraisal of sources of evidence.	N/A
METHODS	13	Describe methods used for handling and summarizing the data.	4
RESULTS	14	Give numbers of sources screened, assessed, and included, with reasons for exclusions.	6-7 (Figure 2)
RESULTS	15	Present characteristics of included sources of evidence.	6-8; Supplementary Table S2
RESULTS	16	If done, present data on critical appraisal of included sources.	N/A
RESULTS	17	Present relevant data charted from included sources.	8-10 (Tables 1-6)
RESULTS	18	Summarize and/or present the charting results.	8-11
DISCUSSION	19	Summarize the main results and link them to the review objectives.	11-13
DISCUSSION	20	Discuss limitations of the scoping review process.	12-13
DISCUSSION	21	Provide a general interpretation of the results and potential implications.	12-14
FUNDING	22	Describe sources of funding for the included sources and the review.	14

Supplementary Table S1 - PRISMA checklist adapted from: Tricco AC, Lillie E, Zarin W, O'Brien KK, Colquhoun H, Levac D, et al. PRISMA Extension for Scoping Reviews (PRISMAScR): Checklist and Explanation. Ann Intern Med. 2018;169:467-473. doi:10.7326/M18-0850.
