# Supplementary material for: Paget–Schrotter Syndrome in the Primary Care Setting: A Case Report and Scoping Review of Clinical Presentation and Diagnostic Delay
Source: Clin Pract. 2026 Sep 19;16(9):172. doi: 10.3390/clinpract16090172 (PMC13604958; doi:10.3390/clinpract16090172)
Supplement: Supplementary file 1 [file clinpract-16-00172-s001.zip › Supplementary Table S2 full literature table hyperlinks PSS 18092026.pdf]

Supplementary table S2 - complete list of included studies with hyperlinks and contributed cases

Name of publication with hyperlink	Year	Cases contributed
Acute axillosubclavian vein thrombosis (Paget-Schroetter syndrome) detected by Tc-99m MAA during pulmonary perfusion scintigraphy.	2000	1
Simultaneous bilateral upper extremity venous thrombosis in a factor V Leiden heterozygote: a case report and review	2001	1
Primary subclavian venous thrombosis which developed after sleeping with the arm in an outstretched position: report of a case	2001	1
Multiple Pulmonary Emboli With Pulmonary Hypertension Caused by Effort Thrombosis and Effective Balloon Venoplasty of the Subclavian Vein	2001	1
Paget-Schroetter syndrome in sports activities--case study and literature review	2001	8
Subclavian vein thrombosis caused by an unusual congenital clavicular anomaly in an atypical anatomic position	2002	1
Effort thrombosis in the elite throwing athlete	2002	4
Effort-induced thrombosis: diagnosis with three-dimensional MR venography	2002	1
Effort thrombosis of the subclavian-axillary vein	2002	7
Paget-Schroëtter syndrome what to do	2003	1
Effort-induced thrombosis of the subclavian vein--a case of Paget-Schroetter syndrome.	2003	1
Paget-Schroetter syndrome in the young and active	2005	1
An unusual swelling in the neck	2005	1
Paget-Schroetter syndrome in an overhead athlete	2005	1
Occupational relevance of subclavian vein thrombosis in association with thoracic outlet syndrome	2005	1
Thoracic outlet syndrome, Paget-Schroetter syndrome and aberrant subclavian artery in a young man	2006	1
Life-threatening acute and chronic thromboembolic pulmonary hypertension and subclavian vein thrombosis	2006	3
Atraumatic Subclavian Vein Thrombosis in a Collegiate Baseball Player: A Case Report	2006	1
Effort thrombosis of the upper extremities related to an arm stretching exercise.	2006	1
Paget-Schroetter syndrome in a collegiate football player.	2006	1
Diagnosis of a rare source of upper extremity symptoms in a healthy woman after weight lifting	2006	1
Effort-induced venous thrombosis of the upper limbs	2006	3
Push-up exercise induced thrombosis of the subclavian vein in a young woman: report of a case	2007	1
Case of adolescent with Paget-Schroetter syndrome and underlying thrombophilia due to an elevated lipoprotein (A)	2006	1
An 18-year-old with effort-related arm swelling.	2007	1
Paget-Schroetter syndrome.	2007	1
Effort thrombosis of the upper extremity in a young sportsman: Paget-Schroetter syndrome	2007	1
Paget-Schroetter syndrome/Paget-Schroetter sendromu	2008	1
A simple case of upper limb venous thrombosis requiring anticoagulation?	2008	1
Upper Limb Deep Vein Thrombosis and Portable Computer Games	2008	1
Unique treatment regimen for effort thrombosis in the nondominant extremity of an overhead athlete: a case report	2009	1
Effort thrombosis presenting as pulmonary embolism in a professional baseball pitcher.	2009	1
Subclavian Vein Thrombosis Extending into the Internal Jugular Vein: Paget-von Schroetter Syndrome.	2009	1
Upper extremity deep vein thrombosis in a 25 year old apparently healthy man	2010	1
Images in clinical medicine. Paget-Schroetter syndrome.	2010	1
Paget-Schroetter syndrome forerunning the diagnoses of thoracic outlet syndrome and thrombophilia	2010	1
Two cases of upper-extremity swelling Paget-Schroetter syndrome and non-Hodgkin lymphoma	2010	1
Paget-Schroetter syndrome: an uncommon cause of pulmonary embolic disease.	2010	1
Work-related effort thrombosis in a millwright: a case report.	2011	1
Effort thrombosis in a young triathlete: an unusual presentation of painless neck swelling secondary to right brachiocephalic vein thrombosis	2011	1
Hand Knitting Induced Thrombosis of the Subclavian Vein in a Young Woman: an Unusual Cause of Paget-Schroetter Syndrome	2011	1
Upper extremity deep vein thrombosis presenting to a chiropractic clinic: a description of 2 cases.	2012	1
Paget Schroetter Syndrome A case study of the chiropractors role in recognizing and comanaging an important condition	2012	1
Upper extremity deep venous thrombosis can you spot the culprit	2012	1
Paget-von Schrötter syndrome: upper extremity deep vein thrombosis after heavy exercise.	2012	1
Paget-Schroetter syndrome diagnosed by bedside emergency physician performed ultrasound a case report	2013	1
Paget-Schrotter syndrome and complications of management	2013	1
Claviculectomy with thrombectomy for management of paget-schroetter syndrome in a patient with chronic clavicular malunion	2013	1
Paget-Schroetter syndrome in a lacrosse player	2013	1
A case report: a young waiter with Paget-Schroetter syndrome.	2013	1

Name of publication with hyperlink	Year	Cases contributed
Vascular thoracic outlet in a competitive swimmer: a case report	2013	1
An unusual presentation of a swollen arm: a case report	2014	1
Thoracic Outlet Syndrome with Secondary Paget Schröetter Syndrome: A Rare Case of Effort-Induced Thrombosis of the Upper Extremity	2014	1
Subclavian vein thrombosis in an otherwise healthy 9-year-old boy	2014	1
Bilateral Upper Extremity DVT in a 43-Year-Old Man: Is It Thoracic Outlet Syndrome?!	2014	1
Effort thrombosis (Paget-Schroetter syndrome) in a 16-year-old male.	2014	1
Paget-Schroetter Syndrome.	2015	1
Upper extremity deep vein thrombosis (Paget-Schroetter syndrome) after surfing: a case report	2015	1
Rock climbing-related subclavian vein thrombosis	2015	2
Upper-limb deep vein thrombosis in Paget-Schroetter syndrome.	2015	1
Acute Pulmonary Embolism due to Paget-Schroetter Syndrome	2015	1
Spontaneous deep vein thrombosis in the upper extremity of a 45-year-old woman	2014	1
Differential diagnosis of a rare case of upper limb pain: Paget-Schroetter syndrome in a doner kebab chef	2015	1
Axillary vein thrombosis induced by an increasingly popular oscillating dumbbell exercise device: a case report	2015	1
Upper extremity deep vein thrombosis in a TV cameraman	2015	1
Combined non-surgical treatment for Paget-Schröetter syndrome: a case report	2016	1
Spontaneous Upper Extremity Venous Thrombosis in a Collegiate Soccer Player: A Case Report	2016	1
A case of Paget-Schroetter syndrome (PSS) in a young judo tutor: a case report.	2016	1
Paget-Schroetter syndrome: diagnostic limitations of imaging upper extremity deep vein thrombosis	2015	1
Painless acrocyanosis: Paget-Schroetter syndrome secondary to thoracic outlet obstruction from muscle hypertrophy	2015	1
Venous Thoracic Outlet Syndrome: The Role of Early Rib Resection	2016	1
Upper Extremity Deep Vein Thromboses: The Bowler and the Barista	2016	1
Upper extremity deep vein thrombosis in a military patient	2015	1
SCREENING FOR REFERRAL BY A SPORTS PHYSICAL THERAPIST REVEALS AN EFFORT THROMBOSIS IN A COLLEGIATE PITCHER: A CASE REPORT.	2016	1
Deep Vein Thrombosis in Upper Limb in a Weightlifter	2017	1
A case of upper extremity deep vein thrombosis with long-term patency using pharmaco-mechanical catheter-directed thrombolysis in the acute phase	2017	1
Paget-Schroetter syndrome in the absence of common predisposing factors: a case report.	2017	1
Paget-Schroetter syndrome complicated by an incidental pulmonary embolism.	2017	1
Young Woman With a Blue, Painful and Swollen Arm	2017	1
Spontaneous Subclavian Vein Thrombosis in a Healthy Adolescent Cheerleader: A Case of Paget-Schroetter Syndrome	2017	1
Bringing to Light Symptoms and Treatments of Effort Thrombosis (Paget-Schroetter Syndrome) in the Military Population, a Case Study	2017	1
Recurrent Upper Extremity Thrombosis Associated with Overactivity: A Case of Delayed Diagnosis of Paget-Schroetter Syndrome	2017	1
Urschel's Sign in Paget Schroetter Syndrome.	2017	1
Diagnosis of Paget-Schroetter Syndrome/Primary Effort Thrombosis in a Recreational Weight Lifter.	2017	1
Paget-Schroetter Syndrome in a Baseball Pitcher.	2017	1
Paget-Schroetter Syndrome Resulting from Thoracic Outlet Syndrome and KAATSU Training.	2017	1
Upper extremity deep vein thrombosis in a triathlete: Again intense endurance exercise as a thrombogenic risk	2017	1
Paget-Schroetter syndrome as a result of 1st rib stress fracture due to gym activity presenting with Urschel's sign - A case report and review of literature	2018	1
Paget-Schroetter syndrome with bilateral pulmonary emboli.	2018	1
Young Man With Left Arm Pain and Swelling	2018	1
A case report on 2 unique presentations of upper extremity deep vein thrombosis	2018	1
Paget-Schroetter syndrome	2018	1
Paget-Schroetter syndrome	2018	1
Sports Injury or Venous Thrombosis?	2019	1
Recurrent pulmonary embolism related with Paget-Schroetter syndrome: a case report	2019	1
Paget-Schroetter syndrome accompanied by pulmonary thromboembolism: A case report.	2019	1
Paget-Schroetter syndrome in a teenager after throwing firecrackers - A case report.	2019	1
Effort Thrombosis Provoked by Saxophone Performance.	2019	1
A swollen arm after backcountry snowboarding in Japan	2019	1
Pulmonary Infarction due to Paget-Schroetter Syndrome and Nephrotic Syndrome	2019	1
A Case of Paget-Schroetter Syndrome in a Young Male After Lifting Weights.	2019	1
Effort Thrombosis in 2 Athletes Suspected of Musculoskeletal Injury	2019	2
Effort Thrombosis Presenting as Unilateral "Pumped" Arm in a Climber.	2020	1

Name of publication with hyperlink	Year	Cases contributed
Venous Thoracic Outlet Syndrome Caused by Double Compression of the Axillosubclavian Vein: A Case Report	2020	1
Paget-Schroetter syndrome in pregnancy: A case report and discussion of management options	2020	1
Arm Pain and Swelling in a College Swimmer: A Case of Paget-Schroetter Syndrome	2020	1
Paget-Schroetter Syndrome: a case report of diagnosis, treatment, and outcome in a healthy 18-year-old athletic swimmer	2020	1
Two Patients with Paget-Schroetter Syndrome That Were Successfully Diagnosed by Doppler Ultrasonography: Case Studies with a Literature Review	2020	1
A Baseball Player with a Swollen Arm	2020	1
Soccer player with unusual right shoulder and arm pain and swelling	2020	1
Upper Extremity Effort Thrombosis	2020	1
Primary upper extremity deep vein thrombosis (effort thrombosis).	2021	1
Paget-Schroetter syndrome in a young fitness enthusiast with a negative D-dimer: highlighting the balance between clinical suspicion and diagnostic modalities.	2021	1
A Case of Upper Limb Venous Thrombosis in Paget-Schroetter Syndrome.	2021	1
Paget Schroetter Syndrome and Chylothorax After Rhinoplasty	2021	1
Delayed Diagnosis of Paget-Schroetter Syndrome in a Patient with COVID-19	2021	1
Paget-Schroetter Syndrome in a Young Female	2021	1
Paget-von Schroetter syndrome: Case report	2021	1
Paget-Schroetter syndrome in a non athlete - a case report	2021	1
Effort-Associated Thrombosis: Imaging Is Key when Suspicion Is High. A Case Report	2022	1
Paget-Schroetter syndrome in an active young female after unsupervised exercise.	2022	1
Venous thoracic outlet syndrome: When exercising may be discouraged	2022	1
Paget-Schroetter Syndrome: A Rare Case of Upper Extremity Deep Vein Thrombosis in a Young Swimmer.	2022	1
Paget-Schroetter syndrome: An unfamiliar cause of upper-limb deep venous thrombosis	2022	1
Paget-Schroetter Syndrome (PSS) and Adjunctive Treatment With Mechanical Aspiration Thrombectomy and Catheter Directed Thrombolysis: A Case Report and Review of Literature	2022	1
Diagnosing Paget-Schroetter Syndrome Using Point of Care Ultrasound (POCUS).	2022	1
Paget-Schroetter syndrome from manual handling at work: Case report and review of the literature	2022	1
Use of MEDEVAC Resources in Austere Settings: Paget-Schroetter in the Deployed Environment	2023	1
A Man in His 50s With an Unusual Network of Small Veins in the Left Infraclavicular Region-Paget-Schroetter Syndrome.	2023	1
Left brachiocephalic venous thrombus initially presenting as acute aortic syndrome	2023	1
Successful paraclavicular surgical decompression for recurrent venous thoracic outlet syndrome following transaxillary first rib resection in an elite athlete	2023	1
Paget-Schroetter syndrome: the importance of early detection and effective surgical intervention.	2023	1
Paget-Schroetter Syndrome: A Case of a Young Weightlifter.	2024	1
Exercise-induced venous thrombosis of the upper extremity: A case report	2024	1
Effort-Induced Deep Vein Thrombosis of the Right Upper Extremity	2024	1
Beyond an upper extremity clot: A case report of paget-schroetter syndrome.	2024	1
Pulmonary embolism following an undiagnosed Paget-Schroetter syndrome: a case report and review of the literature	2024	1
A Rare Cause of Deep Vein Thrombosis in a Young Orchestra Conductor	2024	1
A Case of Paget-Schroetter Syndrome in a Young, Healthy Woman	2025	1
A Case of Paget-Schroetter Syndrome Successfully Treated With Endovascular Treatment	2025	1
Subacute Paget-Schroetter with rapid recurrence and concurrent pulmonary emboli: a case report	2025	1
An Unexpected Complication of Training: A Rare Case of Paget-Schroetter Syndrome in an Adolescent Athlete	2025	1
Thoracic Outlet Syndrome and Thrombosis: A Case of Paget-Schroetter Syndrome Triggered by Prolonged Strap-Holding.	2025	1
